# P-1062. The Role of Infection on Mortality in a Dominican Republic ICU

**DOI:** 10.1093/ofid/ofaf695.1257

**Published:** 2026-01-11

**Authors:** Surelly Mora-Peralta, Patricia Sanrregré, Yeison Reyes-Burgos, Ann S Sánchez-Marmolejos, Rita A Rojas-Fermín

**Affiliations:** Universidad Nacional Pedro Henriquez Ureña, Santo Domingo, Distrito Nacional, Dominican Republic; Universidad Nacional Pedro Henríquez Ureña, Santo Domingo, Distrito Nacional, Dominican Republic; Universidad Nacional Pedro Henriquez ureña, Santo Domingo, Distrito Nacional, Dominican Republic; Hospital General de la Plaza de la Salud, Distrito Nacional, Distrito Nacional, Dominican Republic; Hospital General De La Plaza De La Salud, Distrito Nacional, Distrito Nacional, Dominican Republic

## Abstract

**Background:**

Infections remain a leading cause of morbidity and mortality in intensive care units (ICUs), particularly in low- and middle-income countries where data are limited. This study characterizes infection-related and attributable mortality, pathogens, and risk factors among critically ill patients at the ICU of Hospital General de la Plaza de la Salud (HGPS), Dominican Republic.

**Methods:**

We conducted a retrospective cross-sectional study of ICU patients who died with evidence of infection between May 2022 and May 2023. Data included demographics, infection type, pathogens, ICU length of stay, use of invasive devices, and comorbidities. Deaths were classified as attributable (infection as primary cause) or associated (infection as contributing factor). Descriptive statistics were used.

**Results:**

Among 549 ICU admissions, 101 infection-related deaths were analyzed. Mean age was 60.4 years (18–88), with 52.5% female. Infection was the primary cause in 51.5%. Predominant types—attributable and associated—were bacteremia (18.8%, 15.8%), urinary tract infections (5.9%, 13.8%), pneumonia (2.9%, 3.9%), and skin/soft tissue infections (3.9%, 1.9%). *E. coli* was most frequently isolated (27.7%), followed by *K. pneumoniae* (15.8%) and *P. aeruginosa* (14.8%). Hemodialysis catheters were more common in infection-attributable deaths (27.7%). Major comorbidities included hypertension (77.2%), chronic kidney disease (41.6%), and diabetes (34.7%). Catheter-related bloodstream infections, meningitis, and surgical site infections were linked to prolonged ICU stays.

**Conclusion:**

Infectious diseases were responsible for over half of the ICU deaths at HGPS, with blood infections and urinary tract infections being the most frequent causes, usually linked to the use of invasive devices and ongoing health issues. Bladder catheter use and cardiovascular disease emerged as key modifiable risk factors. These findings highlight the urgent need to improve infection prevention, manage catheters better, and set up strong monitoring systems to lower infection-related deaths in seriously ill patients in places with limited resources.

**Disclosures:**

Rita A. Rojas-Fermín, MD, GSK: Honoraria

